# Investigating the Influence of Heavy Metals and Environmental Factors on Metabolic Syndrome Risk Based on Nutrient Intake: Machine Learning Analysis of Data from the Eighth Korea National Health and Nutrition Examination Survey (KNHANES)

**DOI:** 10.3390/nu16050724

**Published:** 2024-03-02

**Authors:** Seungpil Jeong, Yean-Jung Choi

**Affiliations:** 1 Department of Medical Informatics, College of Medicine, Catholic University of Korea, Seoul 06591, Republic of Korea; seungpil720@catholic.ac.kr; 2 Department of Food and Nutrition, Sahmyook University, Seoul 01795, Republic of Korea

**Keywords:** nutrient intake, environmental exposures, heavy metals, machine learning, phenotypes, metabolic syndrome

## Abstract

This study delves into the complex interrelations among nutrient intake, environmental exposures (particularly to heavy metals), and metabolic syndrome. Utilizing data from the Korea National Health and Nutrition Examination Survey (KNHANES), machine learning techniques were applied to analyze associations in a cohort of 5719 participants, categorized into four distinct nutrient intake phenotypes. Our findings reveal that different nutrient intake patterns are associated with varying levels of heavy metal exposure and metabolic health outcomes. Key findings include significant variations in metal levels (Pb, Hg, Cd, Ni) across the clusters, with certain clusters showing heightened levels of specific metals. These variations were associated with distinct metabolic health profiles, including differences in obesity, diabetes prevalence, hypertension, and cholesterol levels. Notably, Cluster 3, characterized by high-energy and nutrient-rich diets, showed the highest levels of Pb and Hg exposure and had the most concerning metabolic health indicators. Moreover, the study highlights the significant impact of lifestyle habits, such as smoking and eating out, on nutrient intake phenotypes and associated health risks. Physical activity emerged as a critical factor, with its absence linked to imbalanced nutrient intake in certain clusters. In conclusion, our research underscores the intricate connections among diet, environmental factors, and metabolic health. The findings emphasize the need for tailored health interventions and policies that consider these complex interplays, potentially informing future strategies to combat metabolic syndrome and related health issues.

## 1. Introduction

Metabolic syndrome, encompassing a myriad of interrelated conditions, stands as a significant global health threat [1]. This cluster of conditions, which includes elevated blood pressure, heightened blood sugar, abdominal obesity, elevated triglyceride levels, and reduced high-density lipoprotein (HDL) cholesterol levels, presents more than just isolated health issues [2,3]. When viewed collectively, these conditions escalate the risks for severe health complications such as heart disease, stroke, and type 2 diabetes [4]. As individual health concerns, they require rigorous surveillance, but their combined presence indicates potentially dire health trajectories.

The global rise of metabolic syndrome has garnered the attention of significant health organizations, necessitating urgent interventions. The World Health Organization (WHO) and various leading health institutions have documented the escalating prevalence of this syndrome, especially in countries undergoing swift urbanization and experiencing consequential lifestyle shifts [5,6]. This is particularly concerning given the syndrome’s links to chronic diseases, such as heart disease and diabetes, both of which rank highly among global mortality causes [7,8].

Despite the multifaceted origins of metabolic syndrome, ranging from genetics to age-related factors, this research focuses on a more specialized domain: the nexus among dietary patterns and external environmental influences, especially emphasizing the role of heavy metal exposure. Numerous studies have identified a significant association between heavy metals, such as cadmium, mercury, lead, and nickel, and their impacts on nutrient absorption and metabolism [9,10,11,12]. Concurrently, environmental factors, from economic dynamics to individual lifestyle choices like tobacco use and physical activity, have been documented to considerably shape nutritional behaviors and the resultant health outcomes [13,14,15].

The cornerstone of this investigation lies in the exhaustive data provided by the Korea National Health and Nutrition Examination Survey (KNHANES). By leveraging this vast dataset, our intent is to elucidate the multifaceted intersections among dietary habits, environmental variables, and metabolic health. What sets our study apart is our commitment to employing machine learning techniques. Our reliance on machine learning promises a nuanced understanding of dietary and environmental interplays with metabolic syndrome, potentially highlighting correlations that may be understated using conventional research methodologies [16,17,18,19].

This research represents a pivotal exploration into the intricate web of nutrition, environmental factors, and metabolic health. Our approach, combining intensive data scrutiny with state-of-the-art machine learning tools, aspires to furnish insights pivotal for shaping future health interventions and policy-making endeavors.

## 2. Methods

### 2.1. Data Source

Leveraging the vast repository of the Eighth KNHANES, the study aims to delve into the intricate nexus among dietary practices, environmental factors, and metabolic syndrome within the Korean demographic [20]. Renowned for its meticulous aggregation and diverse representation, the KNHANES offers unparalleled insights into Koreans’ evolving health and nutritional paradigms. Its wealth of variables provides granular details on health metrics, nutritional nuances, and ensuing health outcomes, cutting across demographic divisions like age, socio-economic strata, and urban-rural gradients [21]. This breadth ensures an all-encompassing vantage, rendering the dataset indispensable for in-depth public health studies. Tailoring our research focus, we have selectively mined this data, emphasizing variables associated with nutrient intake, heavy metal exposure, and pertinent environmental factors. This pivot is informed by emergent studies indicating potential interconnections among dietary habits, environmental interplay, and metabolic syndrome ramifications [22,23]. Therefore, by harnessing the depth of the KNHANES dataset, we aim to illuminate the intricate interplay between dietary practices, environmental influences, and metabolic health in Korea.

### 2.2. Data Preprocessing

**Figure 1:**
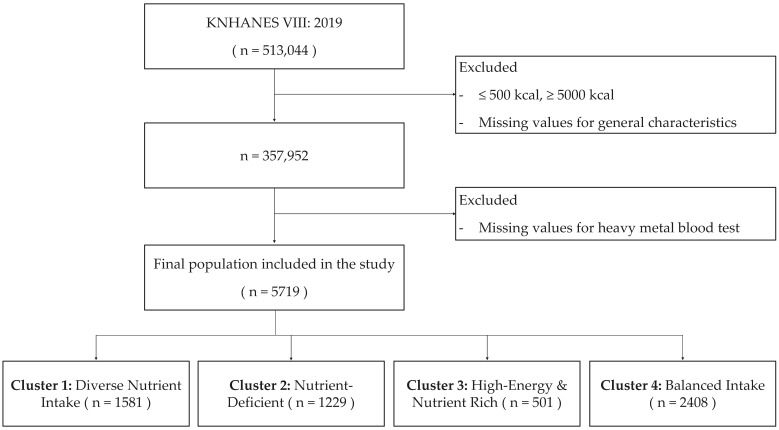
Flowchart that depicts the selection process of study participants.

Associations were analyzed using machine learning techniques on data from the KNHANES. This analysis was conducted on a cohort of 5719 participants who were categorized into four distinct nutrient intake phenotypes (Figure 1). To enhance the fidelity of the Eighth KNHANES for our machine learning analysis, we embarked on a rigorous preprocessing journey. Recognizing the pitfalls of missing data, we employed advanced imputation techniques grounded in probabilistic frameworks to ensure coherent value replacement [24]. Outliers, which can jeopardize model accuracy, were identified and rectified using robust statistical methodologies such as the IQR method and Z-score method [25,26,27,28,29,30]. Furthermore, given the sensitivity of machine learning algorithms to feature scales, normalization processes like Min–Max scaling and Z-score normalization were utilized, ensuring consistent interpretability and optimization across all variables [31,32]. This meticulous refinement transformed the KNHANES dataset into a precision-primed resource, poised to provide robust and insightful analytical outputs.

### 2.3. Machine Learning Approach

Harnessing the power of machine learning in this analytical exploration, the study primarily relied on the K-means clustering algorithm—an unsupervised learning technique celebrated for its precision in parsing multivariate datasets [33,34]. In the expansive realm of nutrition, profiling and categorization can be daunting given the myriad nutritional variables at play. With the KNHANES dataset, this challenge was accentuated, presenting a dense matrix of 25 nutrient dimensions. However, K-means clustering’s adaptability and efficiency proved invaluable, enabling us to succinctly categorize individuals into four distinct nutrient intake profiles or clusters [35,36]. Beyond mere categorization, the application of K-means on this dataset illuminated intrinsic patterns, teasing out the subtle interactions among different nutrient intake, ambient environmental factors, and the resultant metabolic health markers. This clustering exercise further underscored the premise that dietary habits, when viewed through the lens of data-driven algorithms like K-means, can shed light on broader metabolic health trajectories, thereby deepening our understanding of the factors exacerbating or mitigating metabolic syndrome risks [37,38].

### 2.4. Statistical Analyses

Utilizing the robust SAS software (Version 9.4, SAS Institute Inc., Cary, NC, USA), each variable from the Eighth KNHANES was systematically processed using the complex sample design data analysis method, factoring in the survey’s inherent clustering, stratification variables, and weights. Initial descriptive analyses yielded statistics such as means, frequencies, and standard deviations for age and BMI, stratified by nutrient intake clusters. The chi-square test addressed categorical variables, while the analysis of covariance (ANCOVA) was deployed for continuous ones. Furthermore, to identify associations with nutrient intake clusters, we employed univariate logistic regression and expanded upon this with a multiple logistic regression analysis, concentrating on the relationships between participants’ daily dietary nutrient intake and their respective nutrient intake clusters. We maintained a rigorous quality threshold, designating a *p*-value of less than 0.05 as our benchmark for statistical significance.

## 3. Results

### 3.1. General Characteristics of Participants and Their Nutrient Intake Phenotypes

Table 1 provides a comprehensive breakdown of participants by nutrient intake phenotype, offering insights into demographic distribution, health metrics, lifestyle habits, self-reported health status, and education levels. Each cluster presents a unique profile, indicating the diversity and range of characteristics among the participants.

Out of a total of 5719 participants, the average age was 45.70 ± 16.42 years. The distribution among the different nutrient intake phenotype clusters revealed distinct patterns. Cluster 1, consisting of 1581 participants, had an average age of 49.40 ± 16.75 years. Cluster 2 (n = 1229) had an average age of 44.77 ± 17.19 years. Cluster 3 (n = 501) had the youngest participants with an average age of 41.75 ± 14.49 years, and Cluster 4 (n = 2408) had an average age of 44.56 ± 15.76 years. Regarding gender distribution, males represented 39.41% of the total participants, while females represented 60.59%. Gender distribution varied significantly among the clusters.

Metal levels, such as Pb, Hg, Cd, and Ni, were recorded for each participant. The average Pb level was 1.56 ± 0.68 µg/dL for the entire cohort. Cluster-wise, Cluster 3 showed the highest Pb level (1.82 ± 0.29 µg/dL), while Clusters 1, 2, and 4 showed values of 1.44 ± 0.85, 1.46 ± 0.52, and 1.65 ± 0.67 µg/dL, respectively. Similar patterns were observed for Hg, Cd, and Ni.

Health metrics revealed substantial differences among clusters. For obesity, Cluster 3 had the highest proportion of obese participants at 59.48%, whereas Cluster 2 had the lowest at 3.25%. For diabetes, Cluster 3 again had the highest prevalence at 31.54%, while Cluster 4 had the lowest at 4.44%. The distribution for high blood cholesterol, high blood pressure, high fasting blood glucose, and high blood triglyceride followed similar patterns.

Regarding lifestyle habits, there was a notable variance among clusters. For instance, Cluster 3 had the highest proportion of participants eating out daily (77.64%). Cluster 4 had the highest percentage of participants who consume dietary supplements in a year (74.79%). Smoking prevalence was highest in Cluster 4 (16.28%), while Cluster 3 had no current smokers.

Self-reported health and education: Self-reported health status varied among clusters, with Cluster 1 having the most participants reporting good health at 65.46%. On the other hand, Cluster 2 had the highest proportion of participants reporting average or poor health (80.55%). In terms of education, the majority of the participants in Cluster 1 (73.75%) had a high school or lower education.

### 3.2. Characteristics of Heavy Metals and Five Metabolic Syndrome Factors According to Nutrient Intake Level

**Figure 2:**
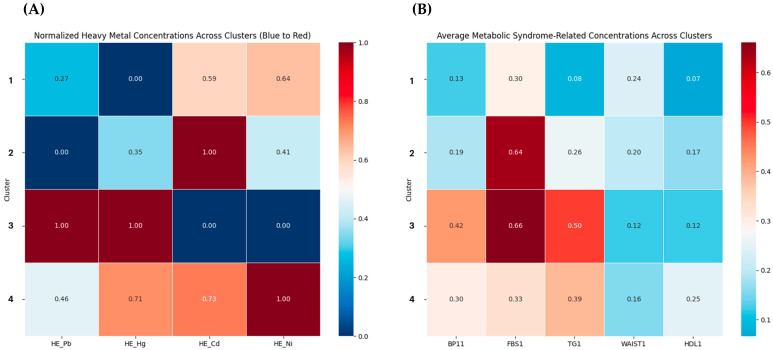
Key results related to clusters. (**A**) Normalized heavy metal concentrations across clusters. (**B**) Average metabolic syndrome-related indicators across clusters.

Diving deep into the multifaceted relationship between heavy metal exposure and metabolic syndrome indicators, our analysis discerned distinct patterns across four specific clusters, each uniquely portraying the interplay between environmental heavy metal exposures and metabolic health outcomes (Table 1 and Figure 2).

Cluster 1 showcased a moderate inclination towards Pb (Lead) exposure, while Hg (Mercury) levels stood out distinctly, overshadowed only by Cluster 3. Interestingly, a spike in cadmium levels was observed here. On the metabolic front, Cluster 1 embodied a moderate hypertension (BP11) level and elevated fasting blood sugar (FBS1)—the latter being second only to Cluster 3. Interestingly, the waist circumference (WAIST1) readings in this cluster hinted at reduced concerns of central obesity as compared to most of its counterparts.

While Cd (Cadmium) exposure echoed patterns seen in Cluster 1, Cluster 2 was characterized by a notable dip in Hg exposure—the lowest among all clusters. Metabolically, this cluster presented a dichotomy: while it showcased the lowest hypertension levels, suggesting better cardiovascular health, it also showed the highest waist circumference and lowest HDL cholesterol (HDL1) levels, implying potential obesity and cholesterol-related challenges.

Alarming insights emerged from Cluster 3. Both Pb and Hg exposures hit peak levels, making this the most exposed cluster. Its metabolic readings resonated with this heightened exposure, revealing peak hypertension and fasting blood sugar levels, flagging substantial cardiovascular and potential diabetes-related risks. In a stark juxtaposition, however, the waist circumference was the lowest.

Cluster 4 reported elevated Pb levels, trailing just behind Cluster 3. Remarkably, this cluster stood out with the highest Ni (Nickel) exposure. In terms of metabolic health, the data painted a mixed picture: while elevated hypertension level readings were a cause for concern, it also showed the peak high-density cholesterol levels, suggesting a relatively healthier cholesterol profile.

### 3.3. Nutrient Intake of Participants across Four Distinct Clusters

In Table 2, each cluster presents distinct dietary patterns, from a diverse nutrient intake to potential deficiencies, to very high-energy consumptions, and finally to a balanced dietary profile.

Individuals in Cluster 1 generally had a broad range of nutrient intakes. They had a significantly higher consumption of energy at 2281.31 kcal, which was above the total average of 1942.76 kcal. Notably, their intake of water, carbohydrates, protein, and fats were all higher than the average, suggesting a possible higher total food consumption or choices dense in these nutrients. The elevated intake of micronutrients like calcium, vitamin A, and vitamin C indicates a potential varied diet.

Cluster 2 showcased the lowest intake across most nutrients, suggesting a potential deficiency or a lower overall food consumption. Their average energy intake was considerably lower at 1235.07 kcal. Micronutrients like calcium, vitamin A, and vitamin C were also consumed in limited amounts. This cluster might be at a nutritional risk and may benefit from interventions to enhance their nutrient intake.

Individuals in Cluster 3 had a remarkable energy consumption, with an average intake of 3165.82 kcal—the highest among all clusters. Their nutrient profile also stands out, with a high intake of proteins, fats, and carbohydrates. The group seemed to prefer diets high in energy, with nutrients like sodium, potassium, and vitamins (like vitamin A and C) being consumed in large quantities.

The largest cluster, Cluster 4, exhibited what can be described as a balanced nutrient intake. Their values generally hovered around the overall averages, suggesting a balanced and potentially healthier diet. While their energy intake was below the overall average, they seemed to have a consistent intake of other nutrients, not veering too high or too low.

### 3.4. Cluster Characteristics across Nutrient Intake Levels

Table 2 provides a breakdown of the average nutrient consumption across four distinct groups of individuals. Under the category of energy and core macronutrients, the table details the intake of energy, water, and basic macronutrients like carbohydrates, proteins, and fats. Specifically, the average caloric intake is represented by the Energy value, where Cluster 3 had the highest consumption, and Cluster 2 the lowest. Water intake, indicating hydration from the diet, also shows that Cluster 3 consumed the most, while Cluster 2 consumed the least. As for carbohydrates, proteins, and fats, Cluster 3 led in consumption, pointing to a high-energy diet.

When considering the types of fat, the table categorizes them into saturated, monounsaturated, and polyunsaturated fatty acids, as well as *n*-3 and *n*-6 fatty acids. Across all fat types, Cluster 3 consumed the most, while Cluster 1 consumed the least. The essential fatty acids, *n*-3 and *n*-6, which are vital for heart health and reducing inflammation, were most consumed by Cluster 3 and least by Cluster 2.

Table 2 further highlights other dietary components like cholesterol and dietary fiber. Cluster 1 had the highest cholesterol intake, closely followed by Cluster 3, which can be a potential health concern. On the other hand, dietary fiber, essential for digestion and often found in whole grains, fruits, and vegetables, was most consumed by Cluster 3. Sugar intake was highest in Cluster 1.

Lastly, under vitamins and minerals, the table lists several essential minerals and vitamins. Except for calcium, Cluster 3 consumed the most of these minerals, with Cluster 1 leading in calcium intake. For the vitamins, Cluster 3 also consumed the most, with the exception of Vitamin C and retinol, where Cluster 1 led.

### 3.5. The Relationship between Various Nutrient Intake Phenotypes and Associated Risk Factors

A distinct association was observed between nutrient intake phenotypes and the levels of various heavy metals (Table 3). The concentration of Pb (µg/dL) showed a significantly high odds ratio in Cluster 3 and 4, with Cluster 3 having an astounding OR of 47.081 (95% CI: 23.867–92.874). This suggests that individuals in the “High-Energy & Nutrient-Rich” cluster may have had higher exposure or retention of lead. Likewise, Hg (µg/L) levels were significantly elevated across all clusters, with the highest being in Cluster 3 at OR: 1.755 (95% CI: 1.567–1.964). Interestingly, Ni concentrations revealed an OR as low as 0.034 in Cluster 4, pointing to a decreased risk.

Heavy drinking and current smoking patterns were predominantly significant risk factors across all nutrient intake phenotypes. For heavy drinking, individuals in the “High-Energy & Nutrient-Rich” cluster (Cluster 3) displayed the least association, with an OR of 0.786. Conversely, individuals in the same cluster who ate out once a day or more were twice as likely, indicating a positive relationship (OR of 2.085). Furthermore, skipping breakfast appeared to be a strong determinant in the “Diverse Nutrient Intake” cluster (Cluster 1), showing a staggering OR of 19.105 for eating breakfast 3–4 times a week.

Household income levels and education seemed to influence nutrient phenotypes. Specifically, those with a college or higher education showed an OR of 0.528 in total, emphasizing the reduced risk among the educated. In terms of economic activity, individuals in the Nutrient-Deficient cluster were over three times more likely to be economically active than the reference group (OR of 2.863).

Physical activity played a pivotal role in shaping nutrient intake. Moderate intensity physical activity showcased protective effects, especially in Cluster 2 and Cluster 4. Walking less than 5 days a week was linked to an increased risk in Cluster 4 (OR: 1.531, 95% CI: 1.317–1.780), emphasizing the importance of regular physical activity for balanced nutrient intake.

## 4. Discussion

### 4.1. Nutrient Level Cluster Analysis

**Figure 3:**
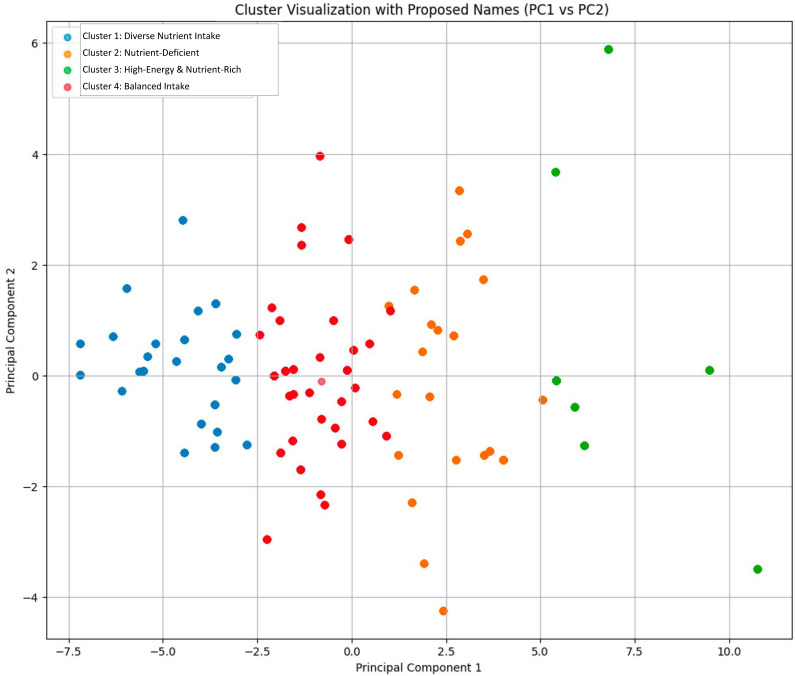
Cluster visualization. This figure showcases the results using the K-means clustering algorithm, which adeptly categorized the data into four discernible nutritional profiles.

This endeavor to demystify nutrient intake and its ramifications on health outcomes required a nuanced approach, given the intricacy of the data spanning 25 diverse nutrient categories. This led us to harness the powers of unsupervised machine learning, precisely, the K-means clustering algorithm [39], which adeptly categorized the data into four discernible nutritional profiles (Figure 3 and Supplementary Figures S1 and S2). Cluster 1 radiated nutritional affluence, endorsing an eclectic array of nutrients. This cohort manifested enhanced levels of energy, fat, saturated fatty acids, cholesterol, dietary fiber, calcium, Vitamin A, and Vitamin C, evocative of a diet dense in fruits, vegetables, dairy, and meats. The salutary effects of such a diverse nutrient palette were echoed by English et al. [40], elucidating the multitude of health boons accompanying such nutritional heterogeneity. Cluster 2 projected nutritional austerity, with conspicuous deficiencies spanning almost all nutrient spectrums. The alarming paucity of energy, fats, carbohydrates, proteins, and an array of vitamins and minerals implies potential malnourishment or a diet predominantly reliant on nutritionally sterile foods. A sustained adherence to such a diet paves the way for a gamut of health pitfalls, a sentiment mirrored by Wang et al. [41]. In Cluster 3, the nutrition profile exudes abundance. Energy, carbohydrates, proteins, varied fatty acids, and a slew of vitamins and minerals flourish in this cluster. While the richness in nutrients promises a slew of health merits, the surging energy content casts shadows of escalating caloric intake. Such surpluses, if not counterbalanced with commensurate physical exertion, can brew health adversities, a notion championed by James Stubbs et al. [42]. Emanating nutritional equilibrium, Cluster 4 shows a balanced act across all nutrients. Unlike its counterparts that exhibited stark nutrient polarities, this group harmoniously aligns moderate nutrient intakes, potentially hinting at a health-optimized diet. The secret lies in striking a balance between portion sizes and ensuring a spectrum of dietary ingredients. Miller et al. [43] accentuate the myriad health dividends sprouting from such balanced indulgences. In essence, these clusters accentuate the complex situation of nutritional intake and underscore the paramountcy of dietary choices in sculpting health destinies.

### 4.2. Analysis of Risk Factors Related to Metabolic Syndrome Using Machine Learning

**Figure 4:**
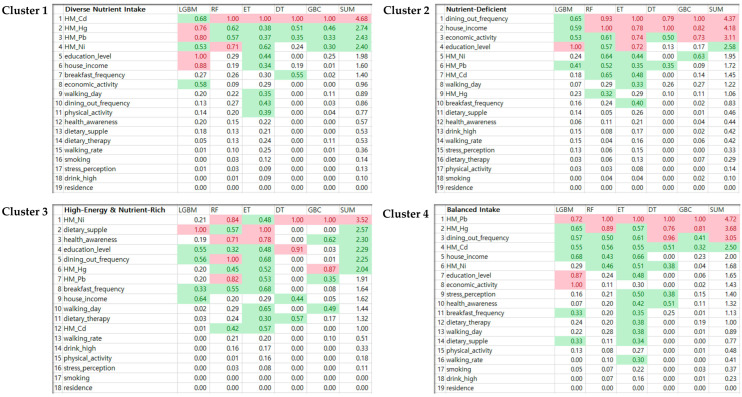
In-depth analysis of the association between heavy metal exposure, environmental variables and their impact on metabolic syndrome. This figure sorts the data into four distinct nutrient intake clusters using the PyCaret 3.1.0 package, an exemplar of automated machine learning. In the figure, different colors represent varying data ranges: red indicates values greater than or equal to 0.7; green shows values between 0.3 and less than 0.7; red (SUM) denotes summed values equal to or exceeding 3; and green (SUM) refers to summed values ranging from 2 to less than 3.

This deep dive into the nexus among heavy metal exposures, environmental variables, and their repercussions on metabolic syndrome crystallized the data into four distinct nutrient intake clusters. The PyCaret 3.1.0 package, epitomizing automated machine learning, streamlined the methodological strides in this study. Among the vast spectrum of 14 classifiers, we gravitated towards five due to their stellar performance (Figure 4 and Supplementary Figure S3). Our choice found its resonance in the seminal work by Kelleher et al. [16], which advocates for uncompromising accuracy and reliability in machine learning tools, particularly when broaching critical health dimensions. Housing participants with a rich and diverse nutrient intake, Cluster 1 registered elevated levels of Cadmium (Cd) and Mercury (Hg). Mehouel and Fowler [44] had earlier walked this path, illuminating the potential dietary origins of such metal exposures. This underscores that while nutrient diversity champions health, it might unwittingly invite heavy metals, thereby mingling benefits with risks. Hallmarked by subpar nutrient intake, Cluster 2 interestingly flagged frequent dining out as a significant behavioral vector. This propensity towards external food sources possibly spells compromised food choices, echoing Chen et al. [45], who espoused the link between certain dining behaviors and nutrient paucity. The recurrent consumption of outside food might inadvertently affect the nutrient equilibrium. The participants in Cluster 3 enjoy energy-laden yet nutrient-rich diets. Their intriguing association with Nickel (Ni) might suggest their exposure to specific foods or environments [46,47]. Aslam et al. [48] have shed light on this interplay, where calorie-rich foods can sometimes be clandestine carriers of heavy metals. This underscores the imperativeness of scrupulous dietary choices even within the realms of nutrient-dense diets. However, the exemplar of dietary equilibrium, Cluster 4, displayed conspicuous Lead (Pb) and Mercury (Hg) levels. Noger-Huet et al. [49] extolled balanced diets without risking potential metal exposures. This observation pivots attention towards the roots of so-called “balanced” diets, raising the question as to whether they inadvertently double as conduits for heavy metal infiltration. These revelations reinforce those diets while being health compasses, and are also cryptic chroniclers of environmental and lifestyle narratives. Each bite not only nourishes but also narrates tales of its origin, travel, and exposures. This encourages a 360-degree purview of diets, embracing their advantages and disadvantages alike. Furthermore, our mathematical modeling, elucidating the significance of classifier outcomes, unraveled a complex set of circumstances binding health, lifestyle markers, and metabolic syndrome. It accentuates the imperativeness of a panoramic view, incorporating both prevention and intervention, in our health journeys. This investigation, in essence, is a clarion call, beckoning a deeper dive into the intriguing interplay of diet, environment, and metabolic health.

### 4.3. Nutrient and Lifestyle Clustering

The meticulous establishment of dietary clusters—“Diverse Nutrient Intake”, “Nutrient-Deficient”, “High-Energy & Nutrient-Rich”, and “Balanced Intake”—unmasked the nuanced interplay between dietary patterns and lifestyle choices. “Diverse Nutrient Intake” mirrors a broad nutritional spectrum, but with potential exposure risks—a sentiment shared by Li et al. [50]. “Nutrient-Deficient” underscores the gaps many face, with their shadowed health ramifications highlighted by Miller et al. [51]. “High-Energy & Nutrient-Rich” presents an opulence of nutrients but with the hidden danger of excess, resonating with the findings of Lachat et al. [52]. Lastly, “Balanced Intake”, celebrated for its moderation, raises questions about its sources and potential contaminants—an insight explored by Mozaffarian and Rimm [53]. These clusters, enriched by the pioneering work of Algur et al. [54], serve not only as dietary compartments but also as illuminative insights into the multifaceted interplay of diet, lifestyle, and health. As elucidated, it is not just about the nutrient profile but the broader context, emphasizing that nutrition’s realm is governed by a careful balance and an understanding of both its gifts and potential pitfalls. Furthermore, the pivotal study by Lind et al. [55] has underscored the potency of such advanced clustering techniques. Their rigorous research accentuates the invaluable role of these techniques in unveiling the complexities of nutrient profiles and their intertwined relationships with metabolic outcomes. Drawing from their insights, it becomes evident that a nuanced understanding of these clusters can be paramount in the development and implementation of precise public health strategies, especially when addressing multifaceted challenges related to metabolic syndrome and associated pathologies.

### 4.4. Underlying Themes in Clusters

The undeniable prominence of heavy metals in all nutritional clusters underscores an omnipresent concern in contemporary nutrition. Whether one’s dietary inclinations lean toward “Diverse Nutrient Intake” or veer toward the “Nutrient-Deficient”, heavy metals have manifested themselves as unyielding components in these dietary categorizations. This all-encompassing significance poses profound questions about the state of our global food chains and offers a stark reminder of the imperatives of monitoring and mitigating heavy metal exposures. The intricate interplay between dietary choices and socio-economic contours emerges as another compelling theme. Specifically, education and income—two crucial pillars of socio-economic status—appear to wield substantial influence over dietary patterns. This correlation implies that individuals’ nutritional choices and the associated risks or benefits might be less about individual discretion and more about broader socio-economic determinants. The frequency of dining out, another lifestyle determinant, surfaces as a critical variable in this narrative. Martins et al. [7] delve deep into this very paradigm, shedding light on the intertwined nature of socio-economic factors, lifestyle choices, and the risk of heavy metal exposures. Such intricate relationships reiterate the necessity for a holistic perspective on nutrition—one that goes beyond mere dietary components and investigates the broader socio-economic and environmental circumstances that shape our food choices.

### 4.5. Implications for Metabolic Syndrome

In our attempt to understand the complexities surrounding metabolic syndrome, the capabilities of machine learning models have proven invaluable. By affording us the ability to parse vast amounts of data and identify intricate patterns, these models have brought forth distinct insights, particularly concerning the omnipresence of certain heavy metals across various nutritional profiles. Whether one’s diet is teeming with nutrients or is conspicuously deficient, the pervasiveness of these metals remains a constant. This ubiquity not only points to the possible health implications of heavy metal exposure but also raises pressing concerns about our larger food supply chain and environment. Such revelations beckon an imperative: the need for an exhaustive investigation into where our foods come from, how they are processed, and what external factors might be contaminating them. This holistic understanding is paramount to framing preventive and therapeutic measures against metabolic syndrome effectively. Deng et al. [56] accentuate this very sentiment. By underscoring the potential of machine learning in elucidating dietary risks for metabolic syndrome, they also touch upon the intertwined nature of nutrition, environment, and the broader societal structures that impact health outcomes. The onus, thus, lies not just in individual dietary choices but in a collective, comprehensive approach to nutrition—one that incorporates technological prowess with traditional nutritional wisdom to ensure holistic health outcomes.

### 4.6. Limitations

In the analysis of the Eighth KNHANES Data, several limitations are acknowledged. One of the key limitations of this study is that while it provides interesting data on the effects of high versus low exposure to metals, it does not establish specific cut-off points for identifying a risk threshold for heavy metal exposure. This highlights the need for future research to define precise risk thresholds that can guide public health recommendations and interventions. Additionally, the study contends with recall bias due to reliance on participants’ self-reported dietary intakes and exposure to environmental factors. The cross-sectional nature of the study captures only a snapshot in time, making it difficult to infer causality among nutrient intake, heavy metal exposure, and metabolic syndrome risk. There is also potential for overfitting in the machine learning models used for analysis, which might limit their generalizability to other populations or datasets. Moreover, the focus on heavy metals might not encompass all influential environmental factors impacting metabolic syndrome. Finally, significant inter-individual variability, influenced by factors such as genetics and gut microbiota, means that metabolic responses to both nutrients and heavy metals can substantially differ among individuals. Acknowledging these limitations is crucial for accurately assessing the health risks associated with varying levels of heavy metal exposure in the diet and for guiding the interpretation of the study’s insights.

### 4.7. Recommendations and Future Directions

This research, utilizing machine learning in conjunction with epidemiological data, offers pivotal insights for healthcare professionals and policymakers. Delving into these intricate patterns presents an irrefutable need for further empirical exploration. Healthcare practitioners can harness these insights to devise personalized nutritional and lifestyle interventions, ensuring that strategies are more attuned to individual health profiles and needs. Peña-Jorquera et al. [57] underscore the importance of such individualized interventions, arguing for a future in public health that leans heavily on data-informed, tailored approaches.

On the policy front, the consistent presence of heavy metals across different nutritional clusters signals a broader systemic issue extending beyond mere dietary choices. Policymakers, informed by such findings, face a pressing mandate to draft regulations that address these environmental contaminants in our food supply. As this study intertwines advanced machine learning techniques with public health revelations, it not only deepens our current understanding but also sets the stage for future research and policy direction. As we navigate this complex interplay of data, nutrition, and health, it is evident that integrated, multi-disciplinary research approaches are paramount in our pursuit of safeguarding public health.

## 5. Conclusions

In conclusion, our study has provided important insights into the relationships among nutrient intake, environmental exposure to heavy metals, and the risk of metabolic syndrome. The consistent presence of heavy metals across different nutritional clusters indicates a systemic issue that transcends individual dietary choices, pointing to a broader concern within the food supply chain. Our findings highlight the critical need for policymakers to consider regulations that address environmental contaminants in our food supply. Additionally, this study underscores the value of integrating advanced machine learning techniques with epidemiological data to uncover complex relationships in public health research. As we move forward, it is clear that an integrated, multi-disciplinary approach to research is essential in our continued efforts to safeguard public health. This study not only deepens our current understanding of these complex interplays but also sets the stage for future research and policy directions in the field of nutrition and environmental health.

## Figures and Tables

**Table 1:** General characteristics of participants by nutrient intake phenotypes.

Variables	Nutrient Intake Phenotypes									
	Total		Cluster 1 ^(1)^		Cluster 2 ^(2)^		Cluster 3 ^(3)^		Cluster 4 ^(4)^	
	(n = 5719)		(n = 1581)		(n = 1229)		(n = 501)		(n = 2408)	
	n (%)	*p*	n (%)	*p*	n (%)	*p*	n (%)	*p*	n (%)	*p*
Age (yrs.) ^(5)^	45.70 ± 16.42	<0.0001	49.40 ± 16.75	<0.0001	44.77 ± 17.19	<0.0001	41.75 ± 14.49	<0.0001	44.56 ± 15.76	<0.0001
Sex		<0.0001		0.0528		<0.0001		<0.0001		<0.0001
Male	2254 (39.41%)		752 (47.56%)		133 (10.82%)		443 (88.42%)		926 (38.46%)	
Female	3465 (60.59%)		829 (52.44%)		1096 (89.18%)		58 (11.58%)		1482 (61.54%)	
Pb (µg/dL) ^(5)^	1.56 ± 0.68	<0.0001	1.44 ± 0.85	<0.0001	1.46 ± 0.52	<0.0001	1.82 ± 0.29	<0.0001	1.65 ± 0.67	<0.0001
Hg (µg/L) ^(5)^	3.27 ± 2.23	<0.0001	3.16 ± 2.69	<0.0001	2.71 ± 1.92	<0.0001	3.77 ± 1.55	<0.0001	3.53 ± 2.09	<0.0001
Cd (µg/L) ^(5)^	0.87 ± 0.56	<0.0001	0.99 ± 0.68	<0.0001	0.84 ± 0.45	<0.0001	0.57 ± 0.51	<0.0001	0.86 ± 0.51	<0.0001
Ni (µg/L) ^(5)^	0.33 ± 0.07	<0.0001	0.32 ± 0.09	<0.0001	0.33 ± 0.06	<0.0001	0.30 ± 0.06	<0.0001	0.35 ± 0.07	<0.0001
Obese		<0.0001		<0.0001		<0.0001		<0.0001		<0.0001
Yes	1194 (20.88%)		573 (36.24%)		40 (3.25%)		298 (59.48%)		283 (11.75%)	
No	4525 (79.12%)		1008 (63.76%)		1189 (96.75%)		203 (40.52%)		2125 (88.25%)	
Diabetes		<0.0001		<0.0001		<0.0001		<0.0001		<0.0001
Yes	646 (11.30%)		276 (17.46%)		105 (8.54%)		158 (31.54%)		107 (4.44%)	
No	5073 (88.70%)		1305 (82.54%)		1124 (91.46%)		343 (68.46%)		2301 (95.56%)	
High blood cholesterol		<0.0001		<0.0001		<0.0001		0.0006		<0.0001
Yes	1715 (29.99%)		473 (29.92%)		293 (23.84%)		212 (42.32%)		737 (30.61%)	
No	4004 (70.01%)		1108 (70.08%)		936 (76.16%)		289 (57.68%)		1671 (69.39%)	
High blood pressure		<0.0001		<0.0001		<0.0001		0.0006		<0.0001
Yes	1369 (23.94%)		293 (18.53%)		80 (6.51%)		212 (42.32%)		784 (32.56%)	
No	4350 (76.06%)		1288 (81.47%)		1149 (93.49%)		289 (57.68%)		1624 (67.44%)	
High Fasting blood glucose		<0.0001		<0.0001		<0.0001		<0.0001		<0.0001
Yes	2485 (43.45%)		1007 (63.69%)		388 (31.57%)		331 (66.07%)		759 (31.52%)	
No	3234 (56.55%)		574 (36.31%)		841 (68.43%)		170 (33.93%)		1649 (68.48%)	
High blood TG ^(6)^		<0.0001		<0.0001		<0.0001		0.8934		<0.0001
Yes	1672 (29.24%)		413 (26.12%)		108 (8.79%)		249 (49.70%)		902 (37.46%)	
No	4047 (70.76%)		1168 (73.88%)		1121 (91.21%)		252 (50.30%)		1506 (62.54%)	
Central obesity		<0.0001		<0.0001		<0.0001		<0.0001		<0.0001
Yes	1060 (18.53%)		321 (20.30%)		310 (25.22%)		61 (12.18%)		368 (15.28%)	
No	4659 (81.47%)		1260 (79.70%)		919 (74.78%)		440 (87.82%)		2040 (84.72%)	
Low HDL ^(7)^ cholesterol		<0.0001		<0.0001		<0.0001		<0.0001		<0.0001
Yes	989 (17.29%)		265 (16.76%)		87 (7.08%)		61 (12.18%)		576 (23.92%)	
No	4730 (82.71%)		1316 (83.24%)		1142 (92.92%)		440 (87.82%)		1832 (76.08%)	
Number of MetS ^(8)^ risk factors		<0.0001		<0.0001		<0.0001		<0.0001		<0.0001
0	1896 (33.15%)		233 (14.74%)		531 (43.21%)		140 (27.94%)		992 (41.20%)	
1	1375 (24.04%)		561 (35.48%)		437 (35.56%)		88 (17.56%)		289 (12.00%)	
2	1261 (22.05%)		623 (39.41%)		247 (20.10%)		54 (10.78%)		337 (14.00%)	
3	1098 (19.20%)		164 (10.37%)		14 (1.14%)		158 (31.54%)		762 (31.64%)	
4	61 (1.07%)		0 (0.00%)		0 (0.00%)		61 (12.18%)		0 (0.00%)	
5	28 (0.49%)		0 (0.00%)		0 (0.00%)		0 (0.00%)		28 (1.16%)	
Heavy drinking		<0.0001		<0.0001		<0.0001		<0.0001		<0.0001
Yes	627 (10.96%)		116 (7.34%)		95 (7.73%)		84 (16.77%)		332 (13.79%)	
No	5092 (89.04%)		1465 (92.66%)		1134 (92.27%)		417 (83.23%)		2076 (86.21%)	
Current smoking		<0.0001		<0.0001		<0.0001		<0.0001		<0.0001
Yes	653 (11.42%)		247 (15.62%)		14 (1.14%)		0 (0.00%)		392 (16.28%)	
No	5066 (88.58%)		1334 (84.38%)		1215 (98.86%)		501 (100.00%)		2016 (83.72%)	
Eating out		<0.0001		<0.0001		<0.0001		<0.0001		<0.0001
≥1 time/d	1526 (26.68%)		495 (31.31%)		374 (30.43%)		389 (77.64%)		268 (11.13%)	
≥1 time/w	3113 (54.43%)		690 (43.64%)		670 (54.52%)		54 (10.78%)		1699 (70.56%)	
<1 time/w	1080 (18.88%)		396 (25.05%)		185 (15.05%)		58 (11.58%)		441 (18.31%)	
Eating breakfast		<0.0001		<0.0001		<0.0001		<0.0001		<0.0001
5–7 times/w	3526 (61.65%)		1093 (69.13%)		605 (49.23%)		270 (53.89%)		1558 (64.70%)	
3–4 times/w	416 (7.27%)		193 (12.21%)		56 (4.56%)		30 (5.99%)		137 (5.69%)	
1–2 times/w	789 (13.80%)		39 (2.47%)		355 (28.89%)		67 (13.37%)		328 (13.62%)	
0 times/w	988 (17.28%)		256 (16.19%)		213 (17.33%)		134 (26.75%)		385 (15.99%)	
Diet therapy		<0.0001		<0.0001		<0.0001		<0.0001		<0.0001
Yes	1550 (27.10%)		559 (35.56%)		119 (9.68%)		188 (37.52%)		684 (28.41%)	
No	4169 (72.90%)		1022 (64.64%)		1110 (90.32%)		313 (62.48%)		1724 (71.59%)	
Eating dietary supplements in a year		<0.0001		0.5974		<0.0001		0.6876		<0.0001
Yes	3331 (58.24%)		801 (50.66%)		483 (39.30%)		246 (49.10%)		1801 (74.79%)	
No	2388 (41.76%)		780 (49.34%)		746 (60.70%)		255 (50.90%)		607 (25.21%)	
Self-reported health status		<0.0001		<0.0001		<0.0001		0.0049		<0.0001
Good	2100 (36.72%)		1035 (65.46%)		239 (19.45%)		282 (56.29%)		544 (22.59%)	
Average or poor	3619 (63.28%)		546 (34.54%)		990 (80.55%)		219 (43.71%)		1864 (77.41%)	
Education level		<0.0001		<0.0001		<0.0001		0.0814		<0.0001
High school or lower	3225 (56.39%)		1166 (73.75%)		696 (56.63%)		270 (53.89%)		1093 (45.39%)	
College or higher	2494 (43.61%)		415 (26.25%)		533 (43.37%)		231 (46.11%)		1315 (54.61%)	
Household income level		<0.0001		0.0009		<0.0001		<0.0001		<0.0001
Low or mid-low	1636 (28.61%)		557 (35.23%)		494 (40.20%)		67 (13.37%)		518 (21.51%)	
Mid-high	2349 (41.07%)		457 (28.91%)		482 (39.22%)		373 (74.45%)		1037 (43.06%)	
High	1734 (30.32%)		567 (35.86%)		253 (20.59%)		61 (12.18%)		853 (35.42%)	
Economic activity		<0.0001		<0.0001		<0.0001		<0.0001		<0.0001
Yes	3728 (65.19%)		1121 (70.90%)		710 (57.77%)		417 (83.23%)		1480 (61.46%)	
No	1991 (34.81%)		460 (29.10%)		519 (42.23%)		84 (16.77%)		928 (38.54%)	
Stress awareness		<0.0001		<0.0001		<0.0001		<0.0001		<0.0001
Low	4430 (77.46%)		1399 (88.49%)		865 (70.38%)		434 (86.63%)		1732 (71.93%)	
High	1289 (22.54%)		182 (11.51%)		364 (29.62%)		67 (13.37%)		676 (28.07%)	
Walking		<0.0001		<0.0001		<0.0001		<0.0001		0.0035
<5 days	2085 (36.46%)		325 (20.56%)		768 (62.49%)		61 (12.18%)		931 (38.66%)	
≥5 days	3634 (63.54%)		1256 (79.44%)		461 (37.51%)		440 (87.82%)		1477 (61.34%)	
Moderate intensity physical activity		<0.0001		<0.0001		<0.0001		<0.0001		<0.0001
Yes	1628 (28.47%)		497 (31.44%)		131 (10.66%)		67 (13.37%)		933 (38.75%)	
No	4091 (71.53%)		1084 (68.56%)		1098 (89.34%)		434 (86.63%)		1475 (61.25%)	
Physical activity		<0.0001		<0.0001		<0.0001		<0.0001		<0.0001
<5 days, 30 min	2337 (40.86%)		445 (28.15%)		830 (67.53%)		61 (12.18%)		1001 (41.57%)	
≥5 days, 30 min	3382 (59.14%)		1136 (71.85%)		399 (32.47%)		440 (87.82%)		1407 (58.43%)	

^(1)^ Cluster 1: Diverse Nutrient Intake; ^(2)^ Cluster 2: Nutrient-Deficient; ^(3)^ Cluster 3: High-Energy & Nutrient-Rich; ^(4)^ Cluster 4: Balanced Intake; ^(5)^ Data were expressed as mean ± SD; ^(6)^ TG: triglyceride; ^(7)^ HDL: high density lipoprotein; ^(8)^ MetS: metabolic syndrome.

**Table 2:** Nutrient intake of participants by phenotypes.

Variables	Nutrient Intake Phenotypes									
	Total		Cluster 1 ^(1)^		Cluster 2 ^(2)^		Cluster 3 ^(3)^		Cluster 4 ^(4)^	
	(n = 5719)		(n = 1581)		(n = 1229)		(n = 501)		(n = 2408)	
	Mean ± SD	*p*	Mean ± SD	*p*	Mean ± SD	*p*	Mean ± SD	*p*	Mean ± SD	*p*
Energy	1942.76 ± 635.85	<0.0001	2281.31 ± 362.45	<0.0001	1235.07 ± 330.48	<0.0001	3165.82 ± 421.43	<0.0001	1827.21 ± 354.82	<0.0001
Water	1024.28 ± 425.29	<0.0001	1318.92 ± 352.61	<0.0001	633.97 ± 287.14	<0.0001	1523.31 ± 258.77	<0.0001	926.22 ± 309.54	<0.0001
Carbohydrate	293.73 ± 98.81	<0.0001	342.17 ± 87.59	<0.0001	207.24 ± 65.99	<0.0001	389.02 ± 100.92	<0.0001	286.24 ± 80.70	<0.0001
Protein	73.14 ± 29.63	<0.0001	85.89 ± 13.58	<0.0001	43.36 ± 14.51	<0.0001	139.21 ± 29.91	<0.0001	66.21 ± 12.46	<0.0001
Fat	47.97 ± 25.71	<0.0001	63.33 ± 17.52	<0.0001	23.94 ± 10.94	<0.0001	94.22 ± 24.50	<0.0001	40.53 ± 15.26	<0.0001
SFA	15.19 ± 8.73	<0.0001	20.90 ± 7.97	<0.0001	7.73 ± 5.42	<0.0001	25.00 ± 9.84	<0.0001	13.21 ± 5.51	<0.0001
MUFA	15.23 ± 9.00	<0.0001	19.35 ± 5.93	<0.0001	7.87 ± 4.23	<0.0001	34.01 ± 8.12	<0.0001	12.38 ± 5.13	<0.0001
PUFA	12.82 ± 8.61	<0.0001	16.34 ± 5.83	<0.0001	6.00 ± 2.60	<0.0001	28.00 ± 14.41	<0.0001	10.82 ± 4.82	<0.0001
N3	2.00 ± 1.56	<0.0001	2.45 ± 1.86	<0.0001	1.07 ± 0.64	<0.0001	3.76 ± 2.03	<0.0001	1.81 ± 1.08	<0.0001
N6	10.78 ± 7.67	<0.0001	13.82 ± 5.31	<0.0001	4.88 ± 2.31	<0.0001	24.30 ± 12.88	<0.0001	8.99 ± 4.34	<0.0001
Cholesterol	257.30 ± 197.97	<0.0001	357.41 ± 236.22	<0.0001	120.44 ± 87.06	<0.0001	356.83 ± 121.84	<0.0001	240.72 ± 176.18	<0.0001
Fiber	26.13 ± 10.87	<0.0001	33.71 ± 9.20	<0.0001	15.33 ± 4.73	<0.0001	43.26 ± 9.20	<0.0001	23.10 ± 5.33	<0.0001
Sugar	63.20 ± 36.52	<0.0001	85.82 ± 40.13	<0.0001	40.01 ± 24.30	<0.0001	84.06 ± 37.56	<0.0001	55.85 ± 27.60	<0.0001
Calcium	558.13 ± 267.47	<0.0001	833.62 ± 241.88	<0.0001	297.38 ± 151.46	<0.0001	681.87 ± 153.30	<0.0001	484.59 ± 147.62	<0.0001
Phosphate	1121.26 ± 399.43	<0.0001	1426.54 ± 194.93	<0.0001	652.43 ± 167.82	<0.0001	1843.68 ± 371.05	<0.0001	1009.82 ± 128.86	<0.0001
Iron	11.85 ± 4.29	<0.0001	14.29 ± 3.47	<0.0001	6.88 ± 2.69	<0.0001	17.27 ± 2.84	<0.0001	11.66 ± 2.79	<0.0001
Sodium	3014.86 ± 1237.48	<0.0001	3696.46 ± 1144.95	<0.0001	2091.83 ± 1133.90	<0.0001	4232.85 ± 923.76	<0.0001	2785.04 ± 917.79	<0.0001
Potassium	2992.26 ± 1120.88	<0.0001	3826.90 ± 913.52	<0.0001	1778.61 ± 481.10	<0.0001	4501.70 ± 1241.59	<0.0001	2749.64 ± 505.69	<0.0001
Vitamin A	389.98 ± 214.61	<0.0001	508.49 ± 193.15	<0.0001	205.77 ± 119.16	<0.0001	633.34 ± 230.26	<0.0001	355.54 ± 163.11	<0.0001
Carotene	3135.18 ± 1994.79	<0.0001	3594.08 ± 1749.21	<0.0001	1704.95 ± 1180.96	<0.0001	5729.98 ± 2428.39	<0.0001	3023.99 ± 1706.27	<0.0001
Retinol	127.94 ± 139.69	<0.0001	208.98 ± 206.79	<0.0001	63.69 ± 51.62	<0.0001	155.85 ± 98.71	<0.0001	101.70 ± 87.37	<0.0001
Vitamin B1	1.35 ± 0.70	<0.0001	1.58 ± 0.47	<0.0001	0.77 ± 0.34	<0.0001	2.45 ± 1.22	<0.0001	1.28 ± 0.42	<0.0001
Vitamin B2	1.59 ± 0.68	<0.0001	2.17 ± 0.42	<0.0001	0.82 ± 0.25	<0.0001	2.72 ± 0.28	<0.0001	1.36 ± 0.31	<0.0001
Niacin	14.59 ± 7.01	<0.0001	16.59 ± 5.33	<0.0001	8.61 ± 3.84	<0.0001	29.21 ± 6.70	<0.0001	13.28 ± 3.78	<0.0001
Vitamin C	74.75 ± 69.55	<0.0001	113.04 ± 78.15	<0.0001	37.36 ± 22.52	<0.0001	110.96 ± 71.90	<0.0001	61.16 ± 63.35	<0.0001

^(1)^ Cluster 1: Diverse Nutrient Intake; ^(2)^ Cluster 2: Nutrient-Deficient; ^(3)^ Cluster 3: High-Energy & Nutrient-Rich; ^(4)^ Cluster 4: Balanced Intake.

**Table 3:** Odds ratio of nutrient intake phenotypes and risk factors of participants.

Variables	Nutrient Intake Phenotypes																			
	Total				Cluster 1 ^(1)^				Cluster 2 ^(2)^				Cluster 3 ^(3)^				Cluster 4 ^(4)^			
	(n = 5719)				(n = 1581)				(n = 1229)				(n = 501)				(n = 2408)			
	OR (95% CI)			*p*	OR (95% CI)			*p*	OR (95% CI)			*p*	OR (95% CI)			*p*	OR (95% CI)			*p*
Pb (µg/dL)	3.388	3.140	3.657	<0.0001	2.053	1.826	2.307	<0.0001	3.171	2.565	3.919	<0.0001	47.081	23.867	92.874	<0.0001	6.047	5.294	6.907	<0.0001
Hg (µg/L)	1.23	1.203	1.257	<0.0001	1.144	1.104	1.185	<0.0001	0.867	0.819	0.918	<0.0001	1.755	1.567	1.964	<0.0001	1.464	1.407	1.523	<0.0001
Cd (µg/L)	1.03	0.948	1.119	0.4875	2.149	1.867	2.474	<0.0001	1.388	1.099	1.752	0.0059	1.705	1.249	2.327	0.0008	0.56	0.483	0.650	<0.0001
Ni (µg/L)	0.087	0.046	0.165	<0.0001	0.734	0.263	2.048	0.5546		-		<0.0001		-		<0.0001	0.034	0.012	0.100	<0.0001
Heavy drinking				<0.0001				<0.0001				<0.0001				0.2614				<0.0001
Yes	2.680	2.306	3.116		2.307	1.618	3.288		2.771	1.878	4.087		0.786	0.516	1.196		3.673	2.934	4.597	
No		1				1				1				1				1		
Current smoking				<0.0001				0.0939												<0.0001
Yes	2.367	2.043	2.742		1.238	0.964	1.590			-				-			2.712	2.213	3.325	
No		1				1				1				1				1		
Eating out				<0.0001				<0.0001				<0.0001				0.3581				<0.0001
≥1 time/d	0.349	0.303	0.402		0.377	0.292	0.486		0.068	0.046	0.101		2.085	1.263	3.443		0.12	0.089	0.162	
≥1 time/w	0.393	0.346	0.445		0.175	0.137	0.224		1.312	0.968	1.777		1.859	0.953	3.626		0.213	0.172	0.262	
<1 time/w		1				1				1				1				1		
Eating breakfast				0.0354				<0.0001				<0.0001				0.9332				<0.0001
5–7 times/w		1				1				1				1				1		
3–4 times/w	1.151	0.960	1.380		2.73	2.034	3.664		19.105	9.568	38.15		0.255	0.128	0.508		0.532	0.385	0.737	
1–2 times/w	0.329	0.284	0.380		0.586	0.325	1.058		0.39	0.303	0.502			-			0.446	0.356	0.558	
0 times/w	0.625	0.550	0.710		19.905	14.52	27.288		0.079	0.053	0.117		0.461	0.317	0.671		0.37	0.298	0.459	
Diet therapy				0.0472				<0.0001				<0.0001				0.7242				0.0097
Yes	1.112	1.001	1.234		0.415	0.342	0.503		3.589	2.515	5.123		1.06	0.767	1.465		1.238	1.053	1.456	
No		1				1				1				1				1		
Eating dietary supplements in a year				<0.0001				<0.0001				0.0247				<0.0001				<0.0001
Yes	1.486	1.351	1.634		0.266	0.219	0.323		0.782	0.632	0.969		3.684	2.647	5.127		3.921	3.254	4.724	
No		1				1				1				1				1		
Self-reported health status																				
Good		1		0.7804		1		<0.0001		1		0.3500		1				1		0.0008
Average or poor	1.014	0.921	1.117		0.502	0.414	0.609		0.883	0.679	1.147			-			1.354	1.135	1.616	
Education level				<0.0001				<0.0001				<0.0001				0.0038				<0.0001
High school or lower		1				1				1				1				1		
College or higher	0.528	0.48	0.581		0.294	0.237	0.364		0.118	0.092	0.151		1.596	1.164	2.190		0.619	0.534	0.717	
Household income level								<0.0001				<0.0001								<0.0001
Low or mid-low		1		<0.0001		1				1				1				1		
Mid-high	0.447	0.399	0.502		0.469	0.371	0.592		0.048	0.035	0.065			-			0.380	0.312	0.464	
High	0.992	0.879	1.119		0.172	0.137	0.218		0.094	0.068	0.131			-			1.776	1.454	2.170	
Economic activity				0.1310				<0.0001				<0.0001				0.2614				<0.0001
Yes	1.078	0.978	1.189		3.312	2.681	4.092		2.863	2.305	3.556		0.786	0.516	1.196		0.712	0.612	0.828	
No		1				1				1				1				1		
Stress awareness				0.0274				0.6566				<0.0001								<0.0001
Low		1				1				1				1				1		
High	1.133	1.014	1.266		1.066	0.803	1.416		0.520	0.411	0.657			-			2.847	2.409	3.365	
Walking				0.0225				<0.0001				<0.0001								<0.0001
<5 days	0.894	0.811	0.984		0.222	0.175	0.282		1.777	1.427	2.212			-			1.531	1.317	1.780	
≥5 days		1				1				1				1				1		
Moderate intensity physical activity				0.0092				<0.0001				<0.0001								<0.0001
Yes	0.872	0.786	0.967		2.144	1.756	2.619		0.295	0.201	0.433			-			0.736	0.633	0.856	
No		1				1				1				1				1		
Physical activity				0.0214				<0.0001				<0.0001								<0.0001
<5 days, 30 min		1				1				1				1				1		
≥5 days, 30 min	1.118	1.017	1.229		3.813	3.083	4.716		0.433	0.343	0.545			-			0.681	0.587	0.790	

^(1)^ Cluster 1: Diverse Nutrient Intake; ^(2)^ Cluster 2: Nutrient-Deficient; ^(3)^ Cluster 3: High-Energy & Nutrient-Rich; ^(4)^ Cluster 4: Balanced Intake.

## Data Availability

The datasets for this study are available in the Korea Centers for Disease Control and Prevention database, accessible via the following URL: https://knhanes.kdca.go.kr/knhanes/sub03/sub03_02_05.do, accessed on 7 December 2023. Individuals, including international researchers who sign up for membership, can utilize the raw data from this website. However, it is important to note that both the data access process and the user manual are provided in Korean.

## Supplementary Materials

The following supporting information can be downloaded at: https://www.mdpi.com/article/10.3390/nu16050724/s1, Figure S1: Numerical Significance Between Clusters Related to Nutrient Variables. This figure provides visual confirmation through a heatmap; Figure S2: Mean Scaled Nutrient Values Across Clusters; Figure S3: Training of Machine Learning Models. The Importance Score (IS), representing the importance of the factors calculated by the model by ISMM, was determined.

## Abbreviations

CI	Confidence interval
IS	Importance scores
ISMM	Importance Score Mathematical Model
KNHANES	Korea National Health and Nutritional Examination Survey
OR	Odds ratio
SD	Standard deviations
