# Supplementary material for: Investigating the Influence of Heavy Metals and Environmental Factors on Metabolic Syndrome Risk Based on Nutrient Intake: Machine Learning Analysis of Data from the Eighth Korea National Health and Nutrition Examination Survey (KNHANES)
Source: Nutrients. 2024 Mar 2;16(5):724. doi: 10.3390/nu16050724 (PMC10934821; doi:10.3390/nu16050724)

Supplementary Figure S1

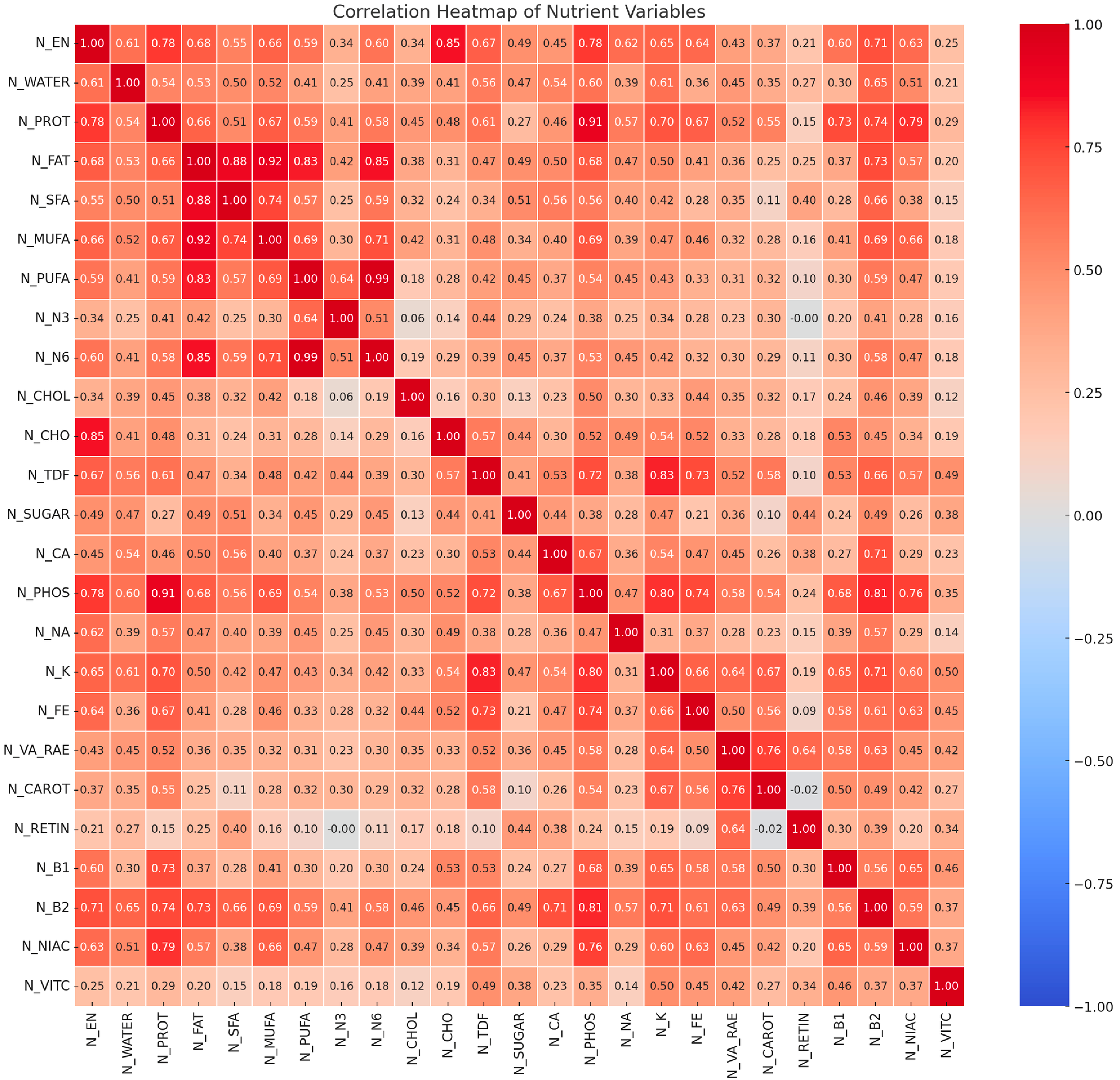

Supplementary Figure S2

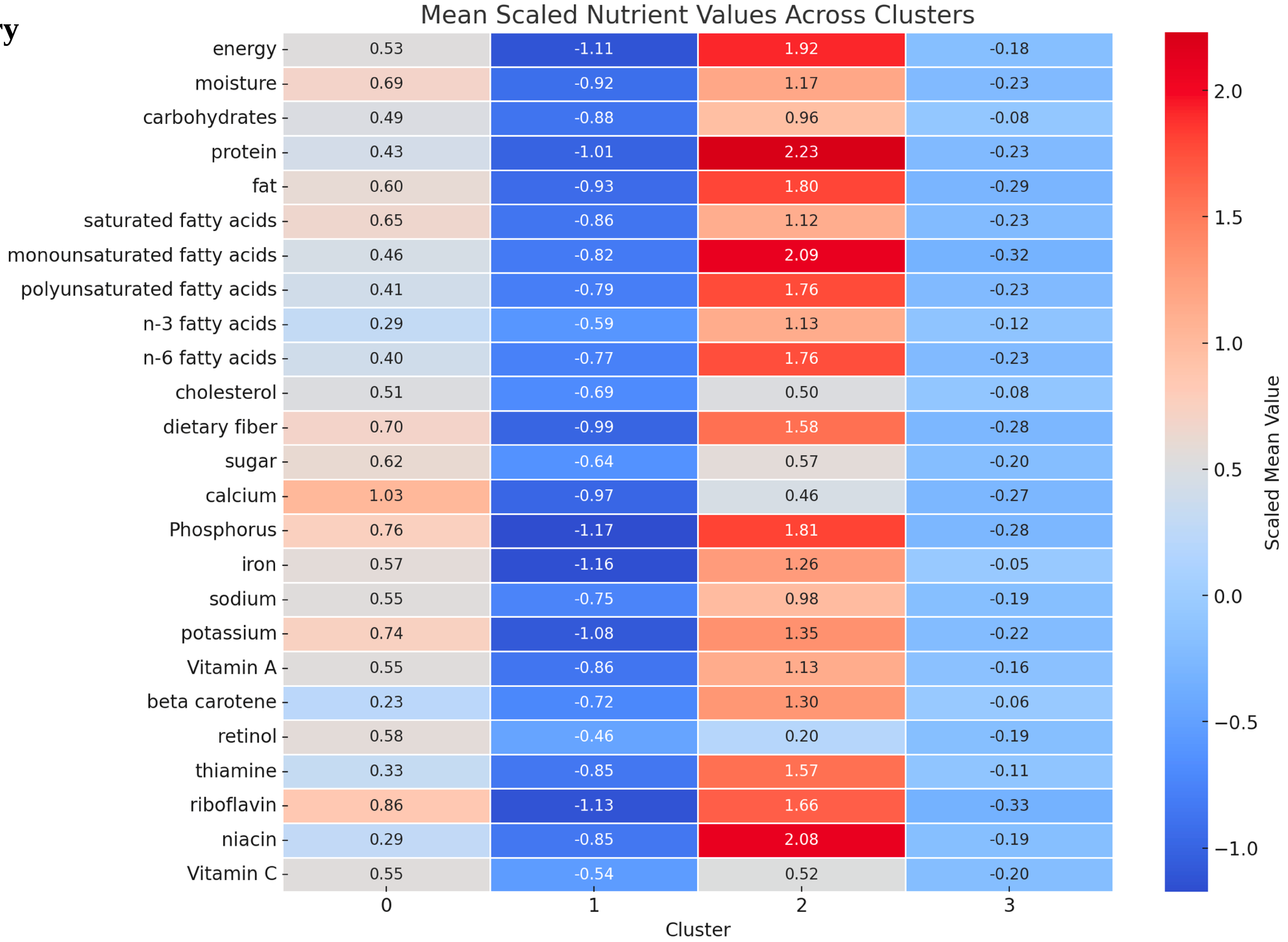

Supplementary Figure S3

Cluster 1

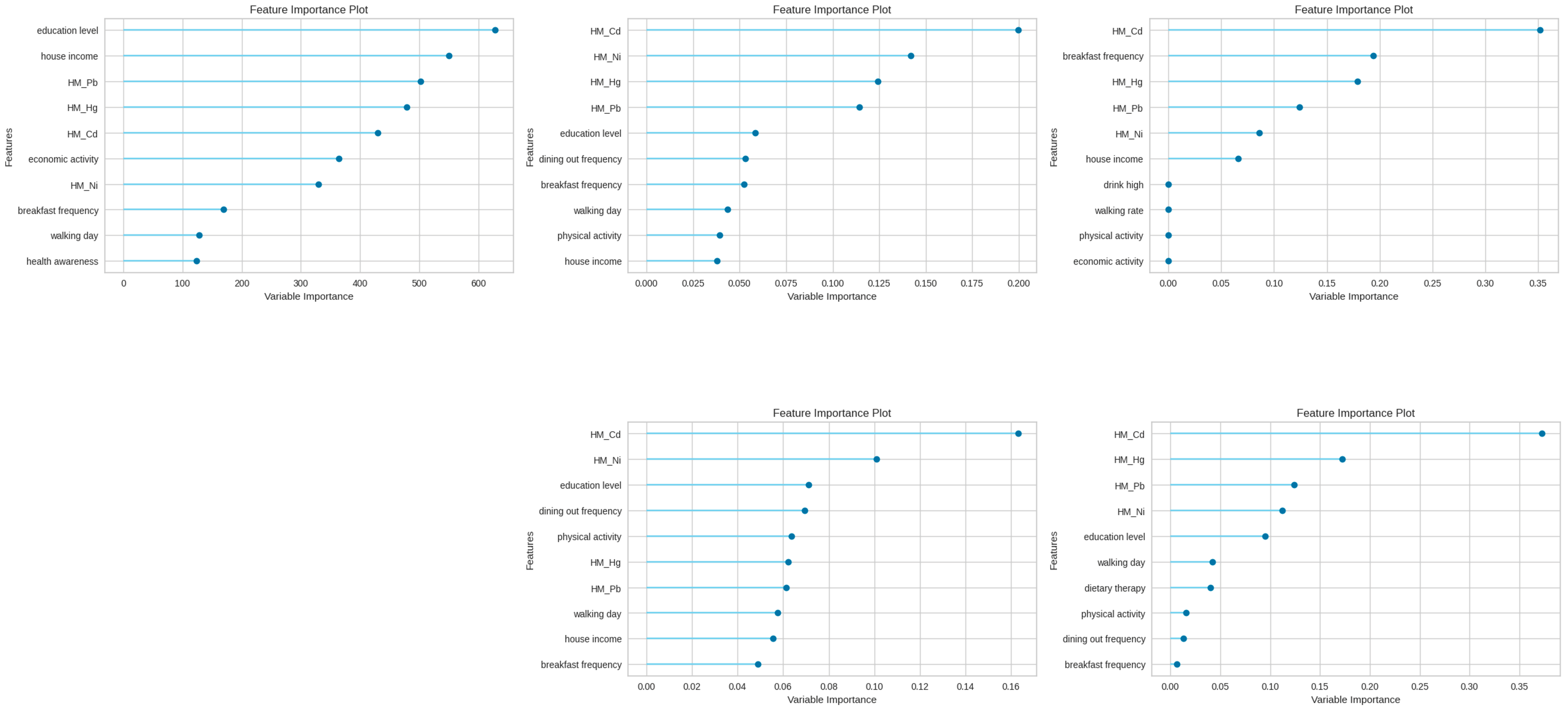

Supplementary Figure S3

Cluster 2

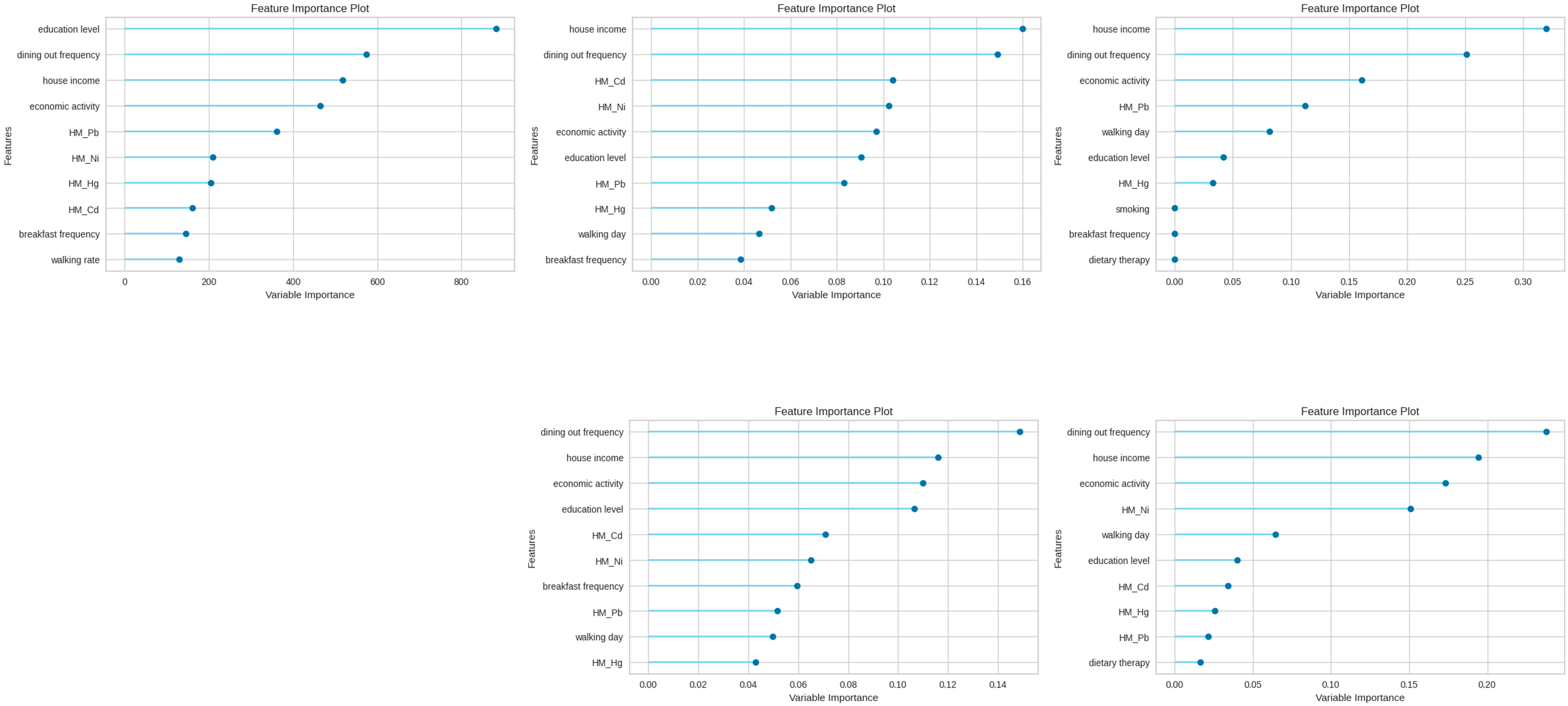

Supplementary Figure S3

Cluster 3

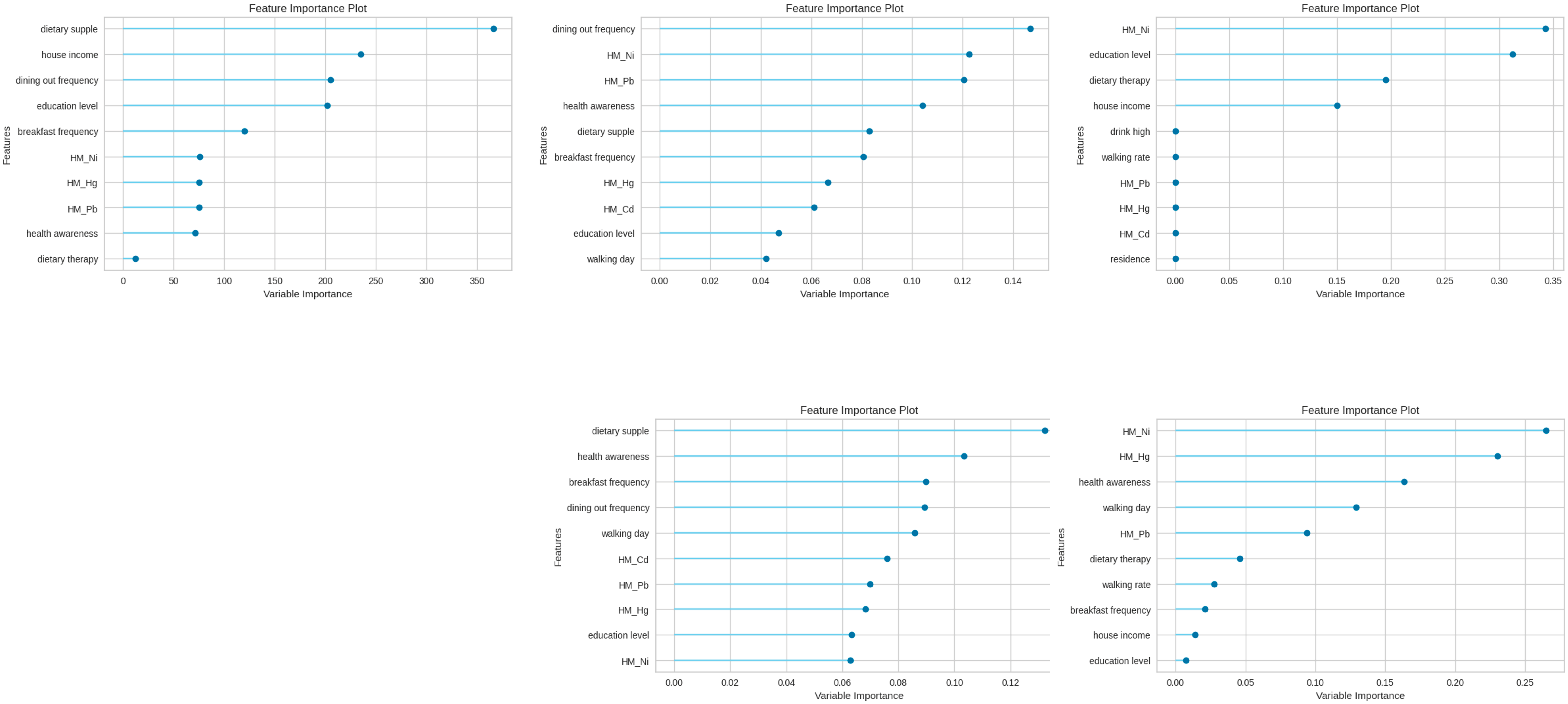

Supplementary Figure S3

Cluster 4

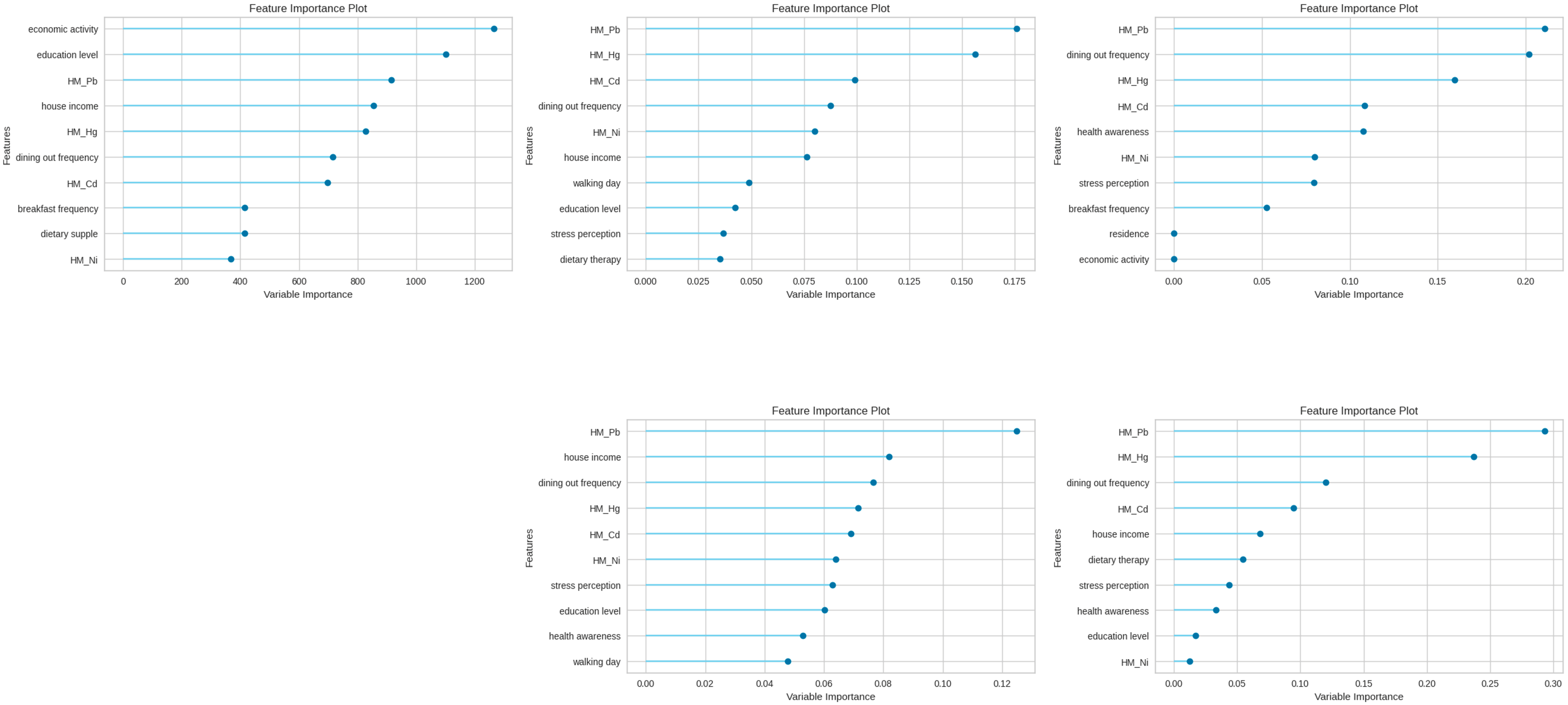

Supplement: Supplementary file 1 [file nutrients-16-00724-s001.zip › nutrients-2870253-supplementary.pdf]
